# The cytotoxic effect and glucose uptake modulation of *Baeckea frutescens* on breast cancer cells

**DOI:** 10.1186/s12906-019-2628-z

**Published:** 2019-08-19

**Authors:** S. H. Shahruzaman, M. F. Mustafa, S. Ramli, S. Maniam, S. Fakurazi, S. Maniam

**Affiliations:** 1 Department of Human Anatomy, Faculty of Medicine and Health Sciences, Universiti Putra Malaysia (UPM), 43400 Serdang, Selangor Darul Ehsan Malaysia; 2 Level 11, Building FF1, Faculty of Pharmacy, UiTM Cawangan Selangor, 42300 Puncak Alam, Selangor Darul Ehsan Malaysia; 3 School of Science, RMIT University, Melbourne, Victoria 3001 Australia

**Keywords:** *Baeckea frutescens*, Glucose uptake, Apoptosis, Oxidative phosphorylation, Breast cancer

## Abstract

**Background:**

*Baeckea frutescens* (*B. frutescens*) of the family Myrtaceae is a plant that has been used in traditional medicine. It is known to have antibacterial, antipyretic and cytoprotective properties. The objective of this study is to explore the mechanism of *B. frutescens* leaves extracts in eliminating breast cancer cells.

**Method:**

*B. frutescens* leaves extracts were prepared using Soxhlet apparatus with solvents of different polarity. The selective cytotoxicity of these extracts at various concentrations (20 to 160 μg/ml) were tested using cell viability assay after 24, 48 and 72 h of treatment. The IC_50_ value in human breast cancer (MCF-7 and MDA-MB-231) and mammary breast (MCF10A) cell lines were determined. Apoptotic study using AO/PI double staining was performed using fluorescent microscope. The glucose uptake was measured using 2-NBDG, a fluorescent glucose analogue. The phytochemical screening was performed for alkaloids, flavonoids, tannins, triterpenoids, and phenols.

**Results:**

*B. frutescens* leaves extracts showed IC_50_ value ranging from 10 -127μg/ml in MCF-7 cells after 72 h of treatment. Hexane extract had the lowest IC_50_ value (10μg/ml), indicating its potent selective cytotoxic activity. Morphology of MCF-7 cells after treatment with *B. frutescens* extracts exhibited evidence of apoptosis that included membrane blebbing and chromatin condensation. In the glucose uptake assay, *B. frutescens* extracts suppressed glucose uptake in cancer cells as early as 24 h upon treatment. The inhibition was significantly lower compared to the positive control WZB117 at their respective IC_50_ value after 72 h incubation. It was also shown that the glucose inhibition is selective towards cancer cells compared to normal cells. The phytochemical analysis of the extract using hexane as the solvent in particular gave similar quantities of tannin, triterpenoids, flavonoid and phenols. Presumably, these metabolites have a synergistic effect in the in vitro testing, producing the potent IC_50_ value and subsequently cell death.

**Conclusion:**

This study reports the potent selective cytotoxic effect of *B. frutescens* leaves hexane extract against MCF-7 cancer cells. *B. frutescens* extracts selectively suppressed cancer cells glucose uptake and subsequently induced cancer cell death. These findings suggest a new role of *B. frutescens* in cancer cell metabolism.

**Electronic supplementary material:**

The online version of this article (10.1186/s12906-019-2628-z) contains supplementary material, which is available to authorized users.

## Background

Survival of patients with metastatic breast cancer has not improved significantly despite the development in early diagnosis and treatments of breast cancer [1]. Multidrug resistance remains the principal obstacle in treating metastatic breast cancer [1].

The inability of cells to respond to stress and repair damage underlies many forms of cancer. A fundamental characteristic of cancer cells is their ability to sustain indefinite cycles of proliferation. One of the mechanism which contributes to the inhibition of tumour growth is by impairing cancer cell metabolism. Reprogramming of core metabolism in tumours confers a selective growth advantage such as the ability to evade apoptosis and/or enhance cell proliferation, promotes tumour growth and progression [2]. The high proliferation rate in cancer cells requires increased energy, and most cancer cells rely on glycolysis as their primary energy source. Pharmacological inhibition of glucose uptake or glycolytic enzymes activity serves as a potential target for cancer therapeutic [3]. To date, natural product plays a dominant role in the discovery of leads for the development of drugs [4].

*Baeckea frutescens* (family Myrtaceae and subfamily Myrtoideae) is a small aromatic low-growing tree found in Peninsular Malaysia, Sumatra, Southern China and Australia [5, 6]. Traditional medicinal properties of *B. frutescens* were reported in Thailand, Malaysia, Sumatra, Borneo, Sulawesi, and New Guinea in treating influenza, dyspepsia, jaundice, dysentery, measles and irregular menstrual cycles [7]. The aromatic property of *B. frutescens* is used to ease mental distress as well as a tonic [8]. Several compounds were isolated from the leaves of *B.frutescens* which include sesquiterpenes [9], phloroglucinols [10–13], chromones [14, 15], flavonoids [16, 17], cyclopentenones and furanones [18], and essential oil [19]. Of note, flavonoid was reported in *B.frutescens* root extracts [20–23]. Cyclopentenones and phloroglucinols were shown to have cytotoxic effect against human pancreatic, lung and breast cancer cell lines [10, 12, 18].

Enhance glycolytic flux in cancer cells contributes to tumour development. One of the mechanism which contributes to the inhibition of tumour growth is by impairing cancer cell metabolism. Thus, it is interesting to explore the potential role of *B. frutescens* in regulating cellular metabolism as an approach in inducing cell death and preventing tumour growth and progression.

## Method

### Extracts preparation

*B. frutescens* or Cucur Atap was collected from Forest Research Institute Malaysia (FRIM) Research Station in Setiu, Terengganu and its voucher specimen was deposited at Institute of Bioscience, Universiti Putra Malaysia (voucher number: KLU 47909). The leaves of *B. frutescens* were air-dried under shade for 7 days and were pulverized into coarse powder [24]. The powder was grounded and filtered using 0.9 mm filter membrane by vacuum pump. The coarse powder was extracted using hexane, ethanol and water. *B.frutescens* leaves ethanol extracts were prepared according to Ahmad et al. [6] at 90% (L90), 70% (L70) and 50% (L50). 111 g, 142 g and 200 g of coarse powder were weighed and extracted in 5 l of ethanol to obtain L90, L70 and L50 respectively. All extraction was prepared by Soxhlet. A stock solution of each crude extract was prepared by suspending 100 mg of extract in 1 mL of pure dimethylsulphoxide (DMSO) and mixed by sonication for 30 min. The volume was adjusted to 1000 mL with culture media to provide assay solutions as required.

### Cell lines

Human MCF-7 and MDA-MB-231 breast carcinoma cells (ATCC, USA) were grown in DMEM supplemented with 100μg/ml streptomycin, 100μg/ml penicillin and 10% FBS. Human mammary breast cell line, MCF10A cells (ATCC, USA) were cultured in DMEM/Ham’s F-12 supplemented with 20 ng/ml epidermal growth factor (EGF), 0.01 mg/ml insulin, 500 ng/ml hydrocortisone and 5% horse serum. All cells were maintained at 37 °C in 5% CO_2._

### Cell viability assay

The cytotoxicity effect of *B. frutescens* leaves extracts was determined by measuring the IC_50_ using cell viability assay as previously described by Mosmann [25]. Briefly, cells at a density of 5 × 10^3^ cells/well were plated in 96-well microplates in triplicates and were treated for 24, 48 and 72 h with extracts concentration ranging from 0 to 1000 μg/ml. Etoposide served as a positive control whilst DMSO was the vehicle control (control). MTT (3-(4,5-dimethylthiazol-2-yl)-2,5-diphenyltetrazolium bromide) solution was added into each well and incubated for 4 h in dark. The formazan grains were dissolved in DMSO and the colour intensity was measured at 570 nm (wavelength range: 550 – 600 nm) using an ELISA plate reader with the reference wavelength of higher than 650 nm.

### Glucose consumption assay

2-NBDG (2-[N-(7-nitrobenz-2-oxa-1,3-diazol-4-yl) amino]-2-deoxy-d-glucose) was used to measure glucose uptake as previously described by Hassanein et al. [26]. Cells were seeded (0–30,000 cells/well) in black flat-bottomed 96-well microplates in triplicates. After overnight incubation, cells were treated with *B.frutescens* leaves extracts for 24, 48 and 72 h. WZB117 (10 μM) served as a positive glucose transport inhibitor and DMSO was used as the vehicle control (control). The cells were incubated with 2-NBDG (100 μM) for 10 min at 37 °C and the reaction was halted by adding two-fold volume of ice-cold PBS. The fluorescent signal was measured using the 485 nm_ex_ and 520 nm_emiss_ filter set.

### Acridine Orange and Propidium iodide staining

Cell death was detected using propidium iodide (PI) (Sigma-Aldrich, USA) and acridine-orange (AO) (Sigma-Aldrich, USA) double staining and examined under fluorescence microscope as previously described by [27]. Briefly, 1 × 10^6^ MCF-7 cells/ml were plated in 6-well plate. The IC_50_ concentration were used for all five extracts and DMSO served as the vehicle control (control). Cells were treated for 24, 48 and 72 h. The treated cells were trypsinized and centrifuged at 1000×*g* for 10 min. After rinsing with PBS, the supernatant was discarded and 10 μl fluorescent dyes, AO (10 μg/ml) and PI (10 μg/ml), were added into the cellular pellet at equal volumes. Stained cells were transferred onto a glass slide and observed under ultraviolet (UV)-fluorescence microscope (Olympus, Japan) within 30 min.

### Phytochemical screening

10 μL of *B.frutescens* leaves extracts (1 mg/mL ethanol) were tested for the presence of tannins, alkaloids, triterpenoids, flavonoids, phenols and alkaloids. The colour intensity was graded as either very high (++++), high (+++), moderate (++), low (+), or not detected (−) compared to the positive control. The quantitative results were determined as previously described by Hisam et al. with slight modification [28]. The absorbance (Wavelength range: 435– 635 nm) was measured by using an ELISA plate reader (Tecan 200, Switzerland).

#### Total tannins content

The intensity of blue colour formation after adding five drops of 5% ferric chloride (Sigma Aldrich) to the extract were measured at 560 nm. 0.01 g/ml tannic acid (Sigma Aldrich) was used as the positive control.

#### Total triterpenoids content

The leaves extracts in chloroform were added with concentrated sulfuric acid and the colour intensity was measured at 635 nm. 0.01 g/ml cholesterol (Sigma Aldrich) was used as the positive control.

#### Total flavonoids content

10% of lead acetate were added to the leaves extracts until yellow precipitate was formed and the colour intensity was measured at 590 nm. 0.01 mg/ml quercetin (Sigma Aldrich) was used as the positive control.

#### Total phenols content

Presence of phenols was measured by adding 10% ferric chloride (Sigma Aldrich) solution to the leaves extracts. The colour intensity was measured at 490 nm. 0.01 g/ml quercetin was used as the positive control.

#### Total alkaloids content

Brown precipitate that was formed when Dragendorff’s reagent (Sigma Aldrich) were added to the extract indicates the presence of alkaloid. The colour intensity was measured at 435 nm. .01 g/ml quinine sulfate (Sigma Aldrich) was used as the positive control.

### Statistical analysis

All experiments were conducted at least three times unless otherwise indicated and the data were expressed as mean ± SEM (Standard Error of the Mean). Statistical analyses were performed using SPSS (version 20) statistical software. The treatment effects were analysed using one way analysis of variance (ANOVA), followed by Bonferroni post-hoc test. *p*-value less than 0.01 (*p* < 0.01) was considered to be statistically significant.

## Results

### Selective cytotoxic effect of *B. frutescens* on breast cancer cells

**Fig. 1:**
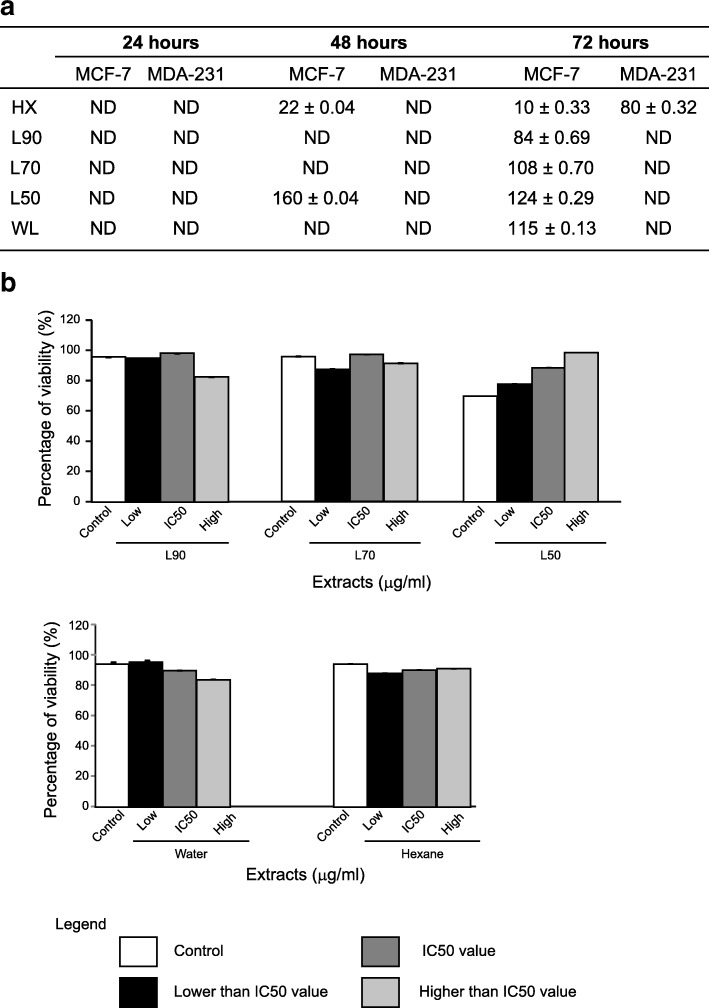
Effects of *B. frutescens* leaves extracts on cell viability. **a** IC_50_ values were determined using cell viability assay in MCF-7 and MDA-MB 231 cells treated with either hexane, L90, L70, L50 or water extracts after 24, 48 and 72 h. **b** Normal mammary epithelial cells (MCF-10A) were treated with either (**a**) L90, L70 or L50 or (**b**) water or hexane extract of *B. frutescens* leaves for 72 h at three different concentrations; lower than IC_50_ value (low), IC_50_ value (IC_50_) and higher than IC_50_ value (high). Data is expressed as mean ± standard error mean based on three independent experiments with triplicate wells for each concentration

**Fig. 2:**
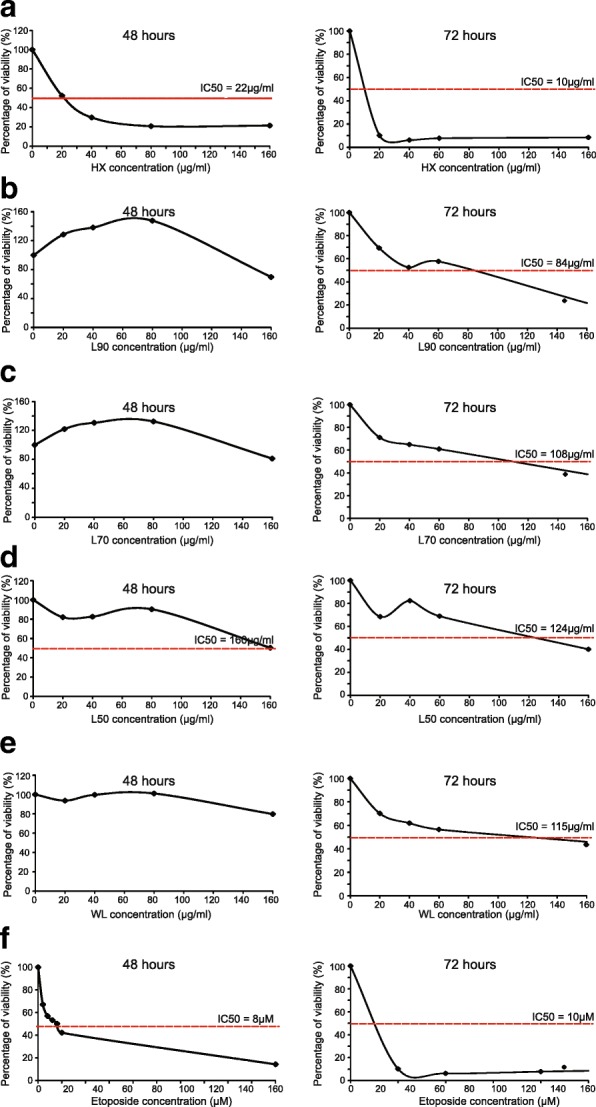
Effects of *B. frutescens* leaves extracts on MCF-7 cell viability. Cytotoxicity was determined using cell viability assay and the IC_50_ value determined as half maximal percentage of cell viability inhibition is indicated by the red horizontal line. MCF-7 cells were treated with either (**a**) hexane, **b** L90, **c** L70, **d** L50 or (**e**) water extract of *B. frutescens* leaves or (**f**) etoposide for 48 (left panels) or 72 (right panels) hrs. Etoposide served as the positive control. Data is expressed as mean ± standard error mean based on six independent experiments with triplicate wells for each concentration

The cytotoxic effect of *B. frutescens* against MCF-7 were determined at 24, 48 and 72 h of incubation. After 24 h of incubation, no cytotoxic effect against MCF-7 cells was observed (Fig. 1a). Interestingly, two extracts (HX and L50) showed IC_50_ values of 22 ± 0.04 μg/ml and 160 ± 0.04 μg/ml, respectively after 48 h of incubation (Figs. 1a and 2). When the incubation time was increased to 72 h, all extracts showed IC_50_ values ranging from 10 to 124 μg/ml (Figs. 1a and 2). Of note, the extracts were also tested against MDA-MB-231 cancer cells at three incubation time; 24, 48 and 72 h. The IC_50_ value of all extracts against MDA-MB-231 cells after 24 and 48 h of incubation cannot be calculated (Fig. 1a). Only hexane extract displayed IC_50_ value of 80 ± 0.32 μg/ml after 72 h of incubation (Fig. 1a). This shows that *B. frutescens* extracts has low cytotoxic effect against MDA-MB-231 compared to MCF-7 cells.

The in vitro cytotoxicity of *B. frutescens* extracts were evaluated in normal mammary epithelial cells, MCF10A. MCF10A cells were incubated in three different concentrations of *B. frutescens* extracts; a concentration lesser than the IC_50_ value obtained from MCF-7 cells, the IC_50_ value and a concentration greater than the IC_50_ value obtained from MCF-7 cells. All extracts except L50 showed 80% of cell viability at their respective IC_50_ values after 72 h of incubation with *B. frutescens* extracts (Fig. 1b).

### Apoptosis induction in breast cancer cells after *B. frutescens* treatment

**Fig. 3:**
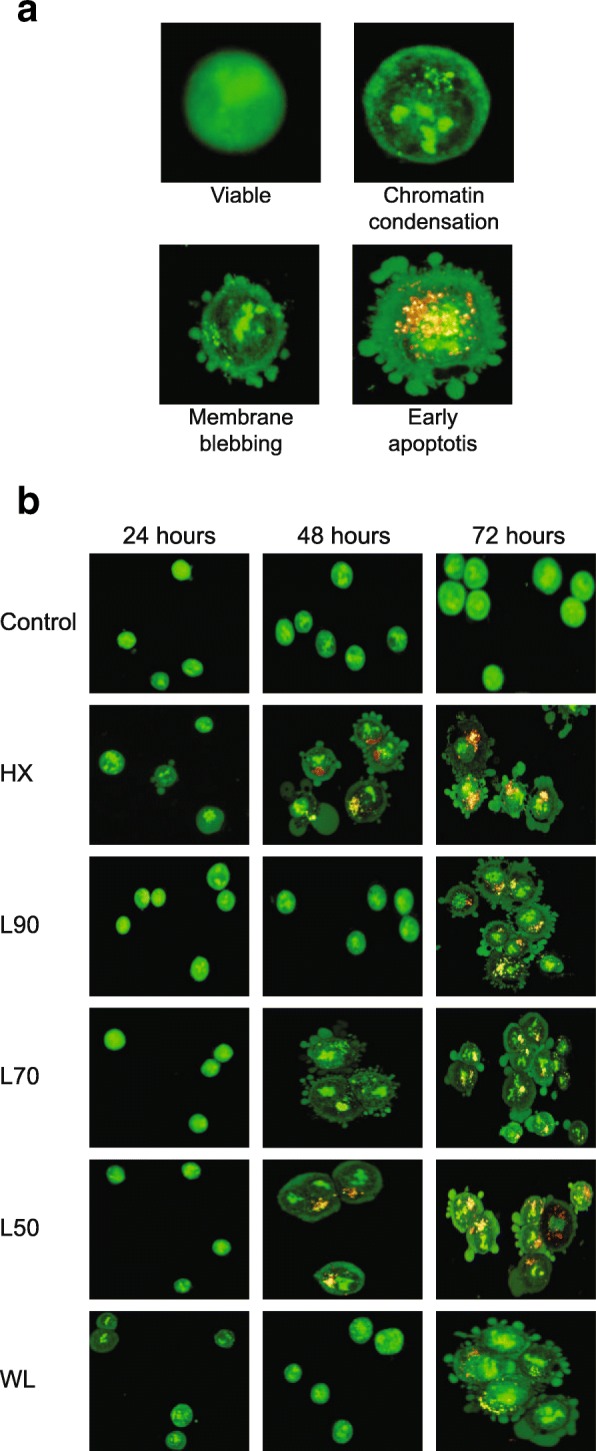
Morphological observation of *B. frutescens* leaves extracts treated MCF-7 cells using AO/PI dual staining at X400 magnifications. **a** Observation of morphological changes in MCF-7 cells. Viable cells are green stained cells with intact nucleus, condensed chromatin marked by intense green stained chromatin, membrane blebbing indicated by the outgrowth of plasma membrane and apoptotic cells are characterised by nuclear disintegration and leakage of plasma membrane. **b** MCF-7 cells were either treated with DMSO vehicle control (control) or *B. frutescens* extracts (hexane, L90, L70, L50 and water) for 24, 48 and 72 h at their respective IC_50_ values

Morphological changes in MCF-7 cells were observed at 24, 48 and 72 h of incubation with five *B.frutescens* extracts at their respective IC_50_ values. At 24 h, the cells were stained green with intact nucleus (Fig. 3b). Interestingly, after 48 h of incubation with HX and L50, chromatin condensation and membrane blebbing were noted (Fig. 3b). As shown in Fig. 3b, cells displayed orange coloured nuclei indicating cell death induction after 72 h incubation with *B.frutescens* extracts.

### Glucose uptake inhibition after *B. frutescens* treatment

**Fig. 4:**
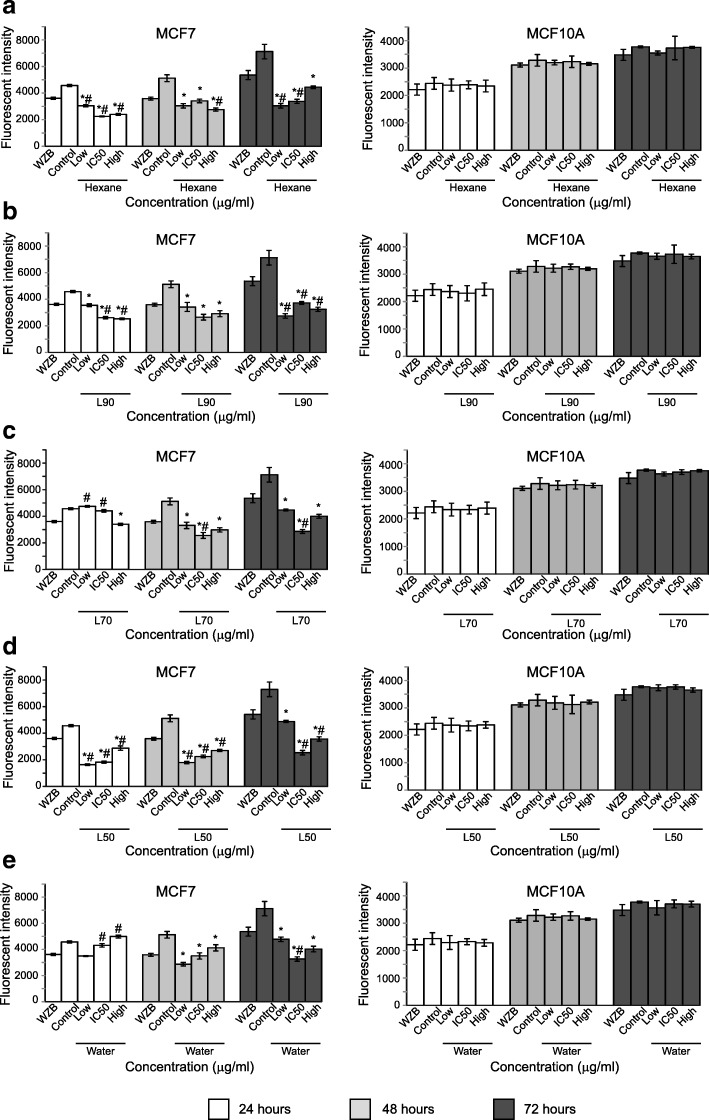
Inhibition of glucose uptake of *B. frutescens* leaves extracts after 24, 48 and 72 h of incubation MCF-7 (left panels) or MCF10A (right panels) cells were either treated with DMSO vehicle control (control) or *B. frutescens* leaves extracts (**a**) hexane, **b** L90, **c** L70, **d** L50 or (**e**) water for 24, 48 and 72 h at three different concentrations; lower than IC_50_ value (low), IC_50_ value (IC_50_) and higher than IC_50_ value (high). WZB117 served as the positive control. Data is expressed as mean ± standard error mean based on four independent experiments with triplicate wells for each concentration. *p < 0.01, compared with control, # *p* < 0.01 compared with the positive control (WZB117)

To investigate the effect of *B. frutescens* extracts on glucose uptake in MCF-7 cells, the cells were incubated in the presence of five extracts at three different concentrations for 24, 48 and 72 h. All extracts except L70 and water extracts, significantly inhibited glucose uptake compared to control at all time points and concentration (Fig. 4).

At 24 h, all extracts except for L70 and water, significantly inhibited glucose uptake compared to the positive control, WZB117 and control at their IC_50_ value. However, only L50 showed significant decrease in glucose uptake compared to WZB117 and control after 48 h of incubation for all three concentrations (Fig. 4d). Interestingly, all extracts at IC_50_ value showed significant decrease in glucose uptake after 72 h of incubation compared to cells treated with WZB117 and control (Fig. 4). Of note, no glucose uptake inhibition was observed in MCF10A treated cells.

### Secondary metabolites identified in leaves and branches of *B. frutescens*

Hexane, ethanol and water extracts showed the presence of various quantities of flavonoids, alkaloids, phenols, triterpenoids and tannins as indicated in Table 1. Alkaloid was undetected in all leaves extracts except very low amount in L90 and L70. Tannin was detected in all five leaves extracts with value of more than 100 (Additional file 1: Table S1). Interestingly, in the qualitative estimation of the secondary metabolites in *B. frutescens* showed that hexane extract has equal ratio of tannin, triterpenoids, flavonoid and phenols (Table 1).

**Table 1:**
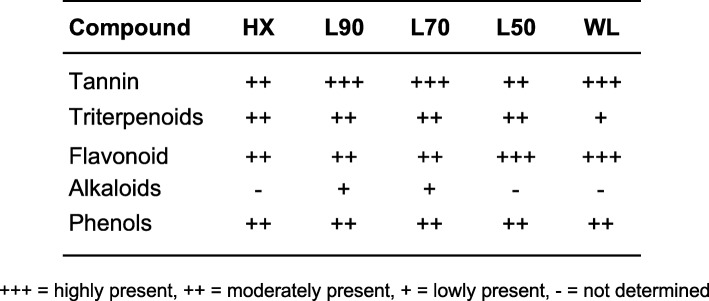
Qualitative analysis of *B. frutescens* leaves phytochemical constituents

## Discussion

Tumours are collection of diverse cells with distinct molecular and morphological signatures [29]. Of note, established biomarkers in breast cancer such as oestrogen receptor, progesterone receptor and HER2 are used in clinical decision-making [30]. In this study, *B. frutescens* extracts were tested in two distinct human breast cancer cell lines: a human breast cancer cell line positive for oestrogen and progesterone receptors and negative for HER-2 receptor and a triple negative cell line, MCF-7 and MDA-MB-231, respectively. MCF-7 cells were found to be more sensitive to *B. frutescens* extracts effect in inducing cell death compared to MDA-MB-231 cells (Fig. 2a). Hence, the subsequent experiments to determine the role of *B. frutescens* in eliminating breast cancer cells were focused on MCF-7 cells.

Cell viability assay and AO/PI staining were used to investigate the cytotoxicity properties of *B. frutescens* leaves extracts on MCF-7 cells. This assay is a non-radioactive measurement of cell viability. Our results indicate hexane extract showed potent selective cytotoxicity against MCF-7 cells at 48 h and 72 h with IC_50_ value less than 20 μg/ml (Figs. 1a and 2). According to the protocol from the American National Cancer Institute (NCI) recommends a crude extract is considered to possess significant cytotoxic activity with IC_50_ value ≤20 μg/ml, whilst this value was deemed at ≤4 μg/ml for a pure compound [31].

The apoptotic effect of these extracts were further established using AO/PI staining where cells predominantly displayed changes that are known to be associated to apoptotic features namely membrane blebbing and chromatin condensation (Fig. 3). Apoptosis is accompanied by series of dramatic cellular morphological changes which includes cell contraction, dynamic membrane blebbing and nuclear disintegration that ultimately lead cells to fragment into apoptotic bodies.

The altered pattern in glucose metabolism in cancer cells was initially described by Otto Warburg as aerobic glycolysis. Hypoxic cancer cells rely heavily on glucose transporters for their survival through the uptake of glucose and subsequent glycolysis. Many cancer cells overexpress GLUT1 [32]. Previous studies on compounds and extracts from *B. frutescens* were tested on cancer cells that exhibit upregulated glucose transporters, mainly GLUT1. Thus, in this study, effect of *B. frutescens* extracts on GLUT1 was investigated.

Glucose is an essential metabolic substrate in cells. GLUT1 is widely distributed in normal tissue and overexpressed in many tumours, including hepatic, pancreatic, breast, oesophageal, brain, renal, lung, cutaneous, colorectal, endometrial, ovarian, and cervical cancers [33]. One major concern about glucose transport inhibitors is the ability to selectively inhibit GLUT1 in tumour but not in normal cells. To address this concern, glucose inhibition assay was performed in the breast cancer cell line MCF-7 and normal mammary cell line MCF10A. This study demonstrated that *B. frutescens* extracts inhibited glucose uptake significantly in cancer cell lines and not in their non-cancerous counterparts (Fig. 4).

To date, knowledge on the mechanism of *B. frutescens* in eliminating cancer cells is limited. This study described a novel role of *B. frutescens* extracts in metabolic reprogramming through their ability in suppressing glucose uptake in cancer cells (Fig. 4). However, the inhibitory effect on GLUT1-mediated glucose uptake may not be the main mechanism in *B. frutescens* in inducing cell death as the inhibition does not correlate with the potency of the extracts which was observed in the MTT assay (Figs. 1, 2 and Table 1). There are several pathways that affect glucose regulation in cancer cells. Disrupting PI3K signalling and selective blocking of GLUT1 transporter lead to decreased glucose uptake by tumours [3, 34]. Ritonavir, fasentine, genistein, STF13 and WZB117 are anticancer drugs designed to target glucose transporter GLUT1 and exert antitumour effect by inhibiting glucose uptake, thus leading to cell death through glucose deprivation [3, 35]. Natural compounds such as cryptocaryone [36, 37], methylxanthines [38] and resveratrol [39] have been recently identified to inhibit glucose uptake either via direct binding to GLUT1 or indirectly by influencing glucose metabolism.

Previous studies have reported on several metabolites extracted from *B. frutescens* and structure of these metabolites were elucidated [5–7, 9–16, 18–24, 40–49]. Prior studies on flavanoids (BF4, BF5 and BF6) [46], phloroglucinols (BF1 and BF2) [10] and tasmanone-based meroterpenoid (Frutescone A –G) [11] extracted from the polar fraction of *B. frutescens* leaves showed moderate cytotoxic activity after 24 h of treatment against leukaemic, hepatocellular carcinoma and colorectal adenocarcinoma cells with IC_50_ ranging from 0.25–50 μM. The studies on phloroglucinols obtained from the non-polar fraction of *B. frutescens* leaves showed cytotoxicity activity against pancreatic, breast and lung cancer cells after 72 h of treatment [12, 44].

The curative properties of medicinal plants are attributed to the presence of various secondary metabolites such as alkaloids, flavonoids, glycosides, natural phenols and terpenoids. In the continuous effort to discover phytochemical compounds from *B.frutescens*, five secondary metabolites, namely tannins, triterpenoids, flavonoids, alkaloids and phenol were tested in the preliminary screening. Hexane is able to extract the most non-polar compounds of all the secondary metabolite. Based on the phytochemical analysis, the active secondary metabolites are of very non-polar in nature, with almost equal amounts of the tannin, flavonoid and phenols. However, the absences of alkaloids is indicative of this nitrogen containing compound is not the active metabolite (Table 1 and Additional file 1: Table S1). Generally, tannin, flavanoid and phenol have been reported for their anticancer properties [50–52]. Potent anticancer activities have been observed in tannins with multiple mechanisms, such as apoptosis, cell cycle arrest, and inhibition of invasion and metastases [51]. This potentially indicates the overall activity of hexane extract is a result of the synergistic combination of the secondary metabolites especially tannin, flavonoid and phenol As the polarity of the solvent is increased from hexane to absolute ethanol, the total amount of triterpenoids, flavonoids and alkaloids increased, as these compounds usually have polar heteroatoms such as nitrogen, oxygen and sulphur. The most polar compounds in the leaves eventually were extracted when water was used as the extracting solvent.

In summary, from the five *B. frutescens* leaves extracts tested, hexane extract showed the most selectively potent cytotoxic activity against breast cancer cell lines, mainly MCF-7 cells. This finding corroborated with the morphological changes observed upon the extract treatment. The hexane extract also selectively inhibited glucose uptake in MCF-7 cells as early as 24 h after treatment.

Cancer is a systemic disease which involves multiple biological network, pathways, genes and proteins. Identification of single compound has shown great success; nonetheless, the effect of single compound is not always ideal [53]. The multiple constituents in *B. frutescens* leaves extracts suggest these extracts may have multiple targets and the efficacy can be achieved by the synergistic and dynamic interaction of multiple constituents. The present study reports the potent cytotoxic effect of *B. frutescens* leaves extracts against breast cancer cells and its novel role in cancer cell metabolism. However, additional studies on synergism and isolation of *B.frutescens* targeting the fractions showing the highest biological activity would be of great interest.

## Conclusions

This study highlights the selective anti-cancer properties of *B. frutescens* in breast cancer cells. MCF-7 cells were more susceptible to *B. frutescens*-mediated cytotoxic effect compared to MDA-MB2–31 cancer cells. The hexane extract showed the most potent effect in inducing cell death with IC_50_ value of less than 20 μg/ml at 48 and 72 h of incubation. Limited publication is available regarding the property of hexane extract isolated from *B. frutescens.* Hence, correlating the potent cytotoxic effect of the hexane extract and its chemical constituents will be pertinent in determining therapeutically active constituent present in *B. frutescens.* Moreover, this study reports the novel role of *B. frutescens* in glucose-uptake inhibition in breast cancer cells. The mechanism of *B. frutescens* extracts in modulating glucose metabolism warrants for further investigation.

## Additional file

Additional file 1: **Table S1.** Phytochemical constituent of *B. frutescens* leaves in 1 μg. A table listing the quantitative of the major secondary metabolites in *B. frutescens* leaves. (DOCX 18 kb)

## Data Availability

The datasets analysed during the current study are available from the corresponding author on reasonable request.

## Abbreviations

2-NBDG: 2-[N-(7-nitrobenz-2-oxa-1,3-diazol-4-yl) amino]-2-deoxy-d-glucose
AO: Acridine-orange
DMEM: Dulbecco’s Modified Eagle Medium
DMSO: Dimethyl sulfoxide
HX: Hexane extract
IC_50_: half maximal inhibitory concentration
L50: *B.frutescens* leaves prepared at the ratio of 1:50 in ethanol
L70: *B.frutescens* leaves prepared at the ratio of 1:70 in ethanol
L90: *B.frutescens* leaves prepared at the ratio of 1:90 in ethanol
MTT: 3-(4,5-dimethylthiazol-2-yl)-2,5-diphenyltetrazolium bromide
PI: Propidium iodide
WL: Water extract
