# Supplementary material for: The cytotoxic effect and glucose uptake modulation of Baeckea frutescens on breast cancer cells
Source: BMC Complement Altern Med. 2019 Aug 19;19:220. doi: 10.1186/s12906-019-2628-z (PMC6700976; doi:10.1186/s12906-019-2628-z)
Supplement: Supplementary file 1 — Table S1. Phytochemical constituent of B. frutescens leaves in 1 μg. A table listing the quantitative of the major secondary metabolites in B. frutescens leaves. (DOCX 18 kb) [file 12906_2019_2628_MOESM1_ESM.docx]

Additional file

**File name**: Additional 1

**Title:** Phytochemical constituent of *B. frutescens* leaves in 1μg

**Description of data:** A table listing the quantitative of the major secondary metabolites in *B. frutescens* leaves

**Table S1 – Phytochemical constituent of *B. frutescens* leaves in 1μg**
